# Application of magnetic cytosmear for the estimation of *Plasmodium falciparum* gametocyte density and detection of asexual stages in asymptomatic children

**DOI:** 10.1186/s12936-016-1170-4

**Published:** 2016-02-24

**Authors:** Deborah Sumari, Brian T. Grimberg, D’Arbra Blankenship, Joseph Mugasa, Kefas Mugittu, Lee Moore, Paul Gwakisa, Maciej Zborowski

**Affiliations:** Bagamoyo Branch, Biomedical Thematic group, Ifakara Health Institute, P.O. Box 54, Bagamoyo, Tanzania; School of Life Sciences and Bioengineering, The Nelson Mandela African Institution for Science and Technology, P.O. Box 447, Arusha, Tanzania; The Centre for Global Health and Disease, Case Western Reserve University, Cleveland, OH 44106-7286 USA; National Institute for Medical Research, Amani Medical Research Centre, P.O. Box 81, Muheza, Tanzania; Muvek Laboratories, P. O. Box 105270, Dar Es Salaam, Tanzania; Department of Biomedical Engineering/ND20, Learner Research Institute, The Cleveland Clinic Foundation, 9500 Euclid Avenue, Cleveland, OH 44195 USA; Genome Science Centre and Department of Veterinary Microbiology and Parasitology, Sokoine University of Agriculture, P.O. Box 3019, Morogoro, Tanzania

**Keywords:** Asymptomatic malaria, MDM, School children, Tanzania

## Abstract

**Background:**

Conventional malaria parasite detection methods, such as rapid diagnostic tests (RDT) and light microscopy (LM), are not sensitive enough to detect low level parasites and identification of gametocytes in the peripheral blood. A modified and sensitive laboratory prototype, Magnetic Deposition Microscopy (MDM) was developed to increase the detection of sub-microscopic parasitaemia and estimation of gametocytes density in asymptomatic school children.

**Methods:**

Blood samples were collected from 303 asymptomatic school children from seven villages in Bagamoyo district in Tanzania. Participants were screened for presence of malaria parasites in the field using RDT and MDM whereas further examination of malaria parasites was done in the laboratory by LM. LM and MDM readings were used to calculate densities and estimate prevalence of asexual and sexual stages of the parasite.

**Results:**

*Plasmodium falciparum* parasites (asexual and sexual stages) were detected in 23 (7.6 %), 52 (17.2 %), and 59 (19.5 %) out of 303 samples by LM, RDT and MDM respectively. Gametocytes were detected in 4 (1.3 %) and 12 (4.0 %) out of the same numbers of samples by LM, and MDM, respectively. Likewise, in vitro results conducted on two laboratory strains of *P. falciparum*, 3D7 and NF54 to assess MDM sensitivity on gametocytes detection and its application on concentrating gametocytes indicated that gametocytes were enriched by MDM by 10-fold higher than LM. Late stages of the parasite strains, 3D7 and NF54 were enriched by MDM by a factor of 20.5 and 35.6, respectively. MDM was more specific than LM and RDT by 87.5 % (95 %, CI 71.2–89.6 %) and 89.0 % (95 % CI 82.9–91.4) respectively. It was also found that MDM sensitivity was 62.5 % (95 % CI 49.5–71.8) when compared with RDT while with LM was 36.5 % (95 % CI 32.2–60.5).

**Conclusions:**

These findings provide strong evidence that MDM enhanced detection of sub-microscopic *P. falciparum* infections and estimation of gametocyte density compared to current malaria diagnostic tools. In addition, MDM is superior to LM in detecting sub-microscopic gametocytaemia. Therefore, MDM is a potential tool for low-level parasitaemia identification and quantification with possible application in malaria transmission research.

## Background

**Fig. 1:**
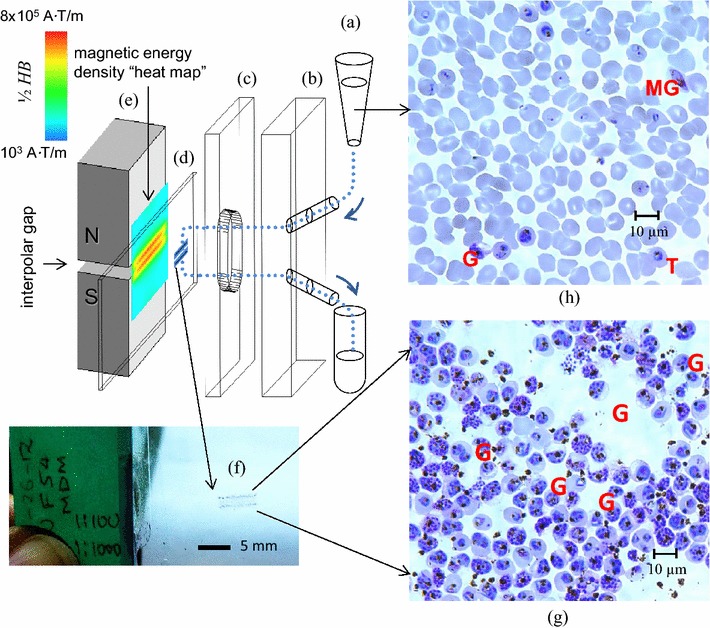
Principle of Magnetic Deposition Microscopy (MDM). The cell suspension (**a**) is pumped through a channel comprising manifold platen with inlet and outlet ports (**b**), channel cut-out spacer (**c**) and a transparent deposition slide (**d**), as indicated by *curved arrows*. The flow channel assembly (**b**–**d**) is pressed against the interpolar gap of a permanent magnet (**e**) generating magnetic force on the magnetically susceptible cells due to a highly non-uniform fringing field, as indicated by the magnetic energy density map (where *H* is the magnetic field strength in amperes per m, A/m, and *B* is the magnetic flux density, in tesla, T). The captured cells form a deposit (**f**) amenable to staining and microscopic analysis. The MDM cell deposit (**g**) shows enrichment in the magnetically susceptible cells compared to the original sample (**h**)—here the malaria parasite-infected erythrocyte culture (NF54 strain, 40× oil magnification). *G* gametocyte, *MG* male gametocyte, *T* trophozoite

The paramagnetic property of biogenic molecules, such as magnetoferritin attached to lymphocytes [1] and haemozoin synthesized inside malaria-infected red blood cells (iRBCs) [2] has been proposed to applications of magnetic separation to clinical research and diagnosis. Malaria infected cells separated from other blood components using magnetic means for medical diagnosis and therapy, have received considerable attention of many investigators [3–5]. In particular, a Magnetic Deposition Microscopy (MDM) device has been developed to capture iRBCs by a high magnetic field gradient in a narrow band on thin polyester (Mylar™) slide (Fig. 1). MDM device operates on the same principle of microscopic examination whereas this concentrates iRBCs to increase sensitivity and decrease the time to read slides compared to conventional light microscopy (LM) [6]. MDM enhances the detection of malaria parasites based on their paramagnetic characteristics due to the presence of intracellular haemozoin [7], a by-product of the parasites’ metabolism after feeding on haemoglobin. *Plasmodium* gametocytes have been reported to produce high haemozoin concentration, sufficient for their detection inside red blood cells using MDM technique [6, 8, 9].

Diagnosis of malaria especially in resource poor settings relies heavily on clinical symptoms or confirmation of *Plasmodium falciparum* parasites in blood by microscopic examination, or by detection of circulating parasite antigens using immunological methods, such as Rapid Diagnostic Test (RDT) [10]. In Tanzania, about 95 % of the communities living at the coast possess mosquito nets and more than half of the households use insecticide-treated nets (ITNs) [11]. Recent malaria survey conducted in Tanzania indicated that interventions have contributed dramatically to a general decline of clinical malaria in Pwani region [12, 13]. This has made it more difficult to diagnose *P. falciparum* at very low levels, hence prompting a shift from focusing only on clinical malaria to identifying and treating asymptomatic malaria infections [14, 15] that sustain transmission.

Malaria transmission depends on several factors, such as presence of mosquito vectors and infectious gametocytes in the peripheral human blood [16–18]. *Plasmodium falciparum* gametocytes circulate in the blood stream for longer period of time than gametocytes of other *Plasmodium* species, and they frequently occur at densities below microscopic detection [19]. Gametocyte detection in the peripheral blood is a good indicator for characterizing the level of transmission [20]. Sub-microscopic infections, especially gametocytaemia, constitute an important parasite reservoir in endemic populations [21] and has become a focus of interest for successful malaria elimination and eradication [22]. A number of studies have been conducted focusing on treating asexual parasites responsible for clinical manifestation of the disease. Little attention has been directed on gametocytes, which are the parasite stage responsible for transmission of the disease even when present at low densities. This study focused on identifying and quantifying infectious reservoirs that sustain malaria transmission and investigated the usefulness of the MDM when used singly or in combination with conventional diagnostic methods in detecting *P. falciparum* gametocytaemia and asymptomatic infections in school-aged children in Bagamoyo district in Pwani region. The MDM device was evaluated firstly, for sensitivity to gametocytaemia and secondly, for ease of use in field operation. This is the first study in Tanzania reporting sensitivity and specificity of MDM for field detection of sub-microscopic infections of *P. falciparum.*

## Methods

### Study design

This was a cross-sectional study conducted in primary school children selected from five schools in two wards in Bagamoyo district, Tanzania. The study was carried out during a high malaria transmission seasons from May to June and from October to December 2014. Prior to commencement of the study, sensitization meetings were held in selected communities and their respective schools. School-teachers helped to distribute consent forms to the participants and arrange meetings with the children’s parents and/or guardians.

A total of 500 school children aged 6–14 years (whose malaria status was unknown) were enrolled of which 303 consented and were recruited for study. Out of the 303 children, 40 were recruited from Kongo primary school in Yombo ward (approximately 25 km from the laboratory) and 136, 49, 43 and 35 children were recruited, respectively from Kidomole, Fukayosi, Mwavi and Msinune primary schools in Kiwangwa ward (approximately 45 km from the laboratory). All 303 children whose parents consented were screened for malaria using RDT and MDM on the spot at the school premises. At the end of screening, the remaining blood was taken to the laboratory for microscopy on the same day. Both symptomatic and asymptomatic malaria cases were referred and offered appropriate treatment at nearby health facilities, according to the national malaria treatment guidelines [23, 24]. The inclusion criteria were child’s asymptomatic status, age between 6–14 years and parent’s consent.

### Ethical consideration

This study received ethical approval from Institutional Review Board (No.IHI/IRB/No: 34-2013) of Ifakara Health Institute and Medical Research Coordinating Committee of the National Institute for Medical Research No. (NIMR/HQ/R.8a/Vol. IX/1705). Regional, district and community authorities in the study area were contacted and granted approval of the study. Prior to participation, a written consent of each respondent was obtained based on Informed Consent Forms (ICFs) designed for the study. Each parent or guardian was given the form that explained risks, benefits and confidentiality that would have been taken. Additionally, the confidentiality of all participants was assured by using unique identity study numbers. The parents and/or guardians, who consented, signed the ICFs and returned back to the study team through head teachers of the schools under study.

### Blood sampling procedures and malaria screening

Three millilitres of venous blood from each participant were collected into heparin vacutainer tubes (Greiner Bio-One GmbH, Kremsmuenster, Austria) for RDT, LM and MDM. The RDT (ICT Malaria Dual Cassette Test; ICT Diagnostics, Cape Town, South Africa) was performed in the field for quick malaria screening as described elsewhere [25]. A volume of 6 and 3 µL of blood were used for thick and thin blood smears immediately after collection respectively. Parasites were counted in accordance to World Health Organization (WHO) standards to detect and quantify parasites and their stages [26]. Fifty microliter of whole blood was diluted 10× with warm phosphate buffered saline (PBS) (37 °C) to final volume of 0.5 mL and used for the MDM. The tubes containing a mixture of blood sample and buffer were kept at 37 °C before running into the prototype. All samples and buffers were maintained at 37 °C.

As a quality control measure two microscopists examined slides independently. Slides with high discordance were reexamined by a third microscopist. Samples were considered negative if no parasites were detected in 100 high-power fields of Giemsa-stained thick blood smears. Both, asexual and sexual stages of the parasites were assessed in thick smears by comparing ratio of infected red blood cells to uninfected ones. Thin smears were examined for parasite quantification. Gametocytes and asexual stage parasites were counted against 500 and 200 white blood cells (WBCs), respectively and densities (parasite per microlitre) were estimated using a factor of 8000 leukocytes/µL.

### In vitro cultivation of malaria parasites

Prior to deployment of MDM for field-testing, its performance was validated and proven using *P. falciparum* laboratory strains, NF54 and 3D7. In vitro cultivation of these two strains was done at Case Western Reserve University, USA. The asynchronous parasite culture was set up at 0.5 % parasitaemia, 4 % haematocrit and maintained using complete malaria culture media [7, 27]. To obtain gametocytes, the cultures were left undisturbed for 4 days to allow induction of gametocytes. The young gametocytes were left to mature by changing the media daily while maintaining 1 % haematocrit. Mature gametocytes developed 15 days later. From the parasite culture stock solution of 1:10, MDM working solution was prepared by 1:10 serial dilution using 1× phosphate buffered saline (PBS) to obtain a working concentration of 1: 1000 dilutions for easy cell examination. The slides were Giemsa-stained and quantified under the microscope by counting 2000 total red blood cells (RBCs) according to WHO guidelines and distinguishing the number of infected and uninfected cells to determine parasitaemia. The slide reading results were recorded on an Excel sheet for further analysis.

### Magnetic deposition microscopy (MDM)

**Fig. 2:**
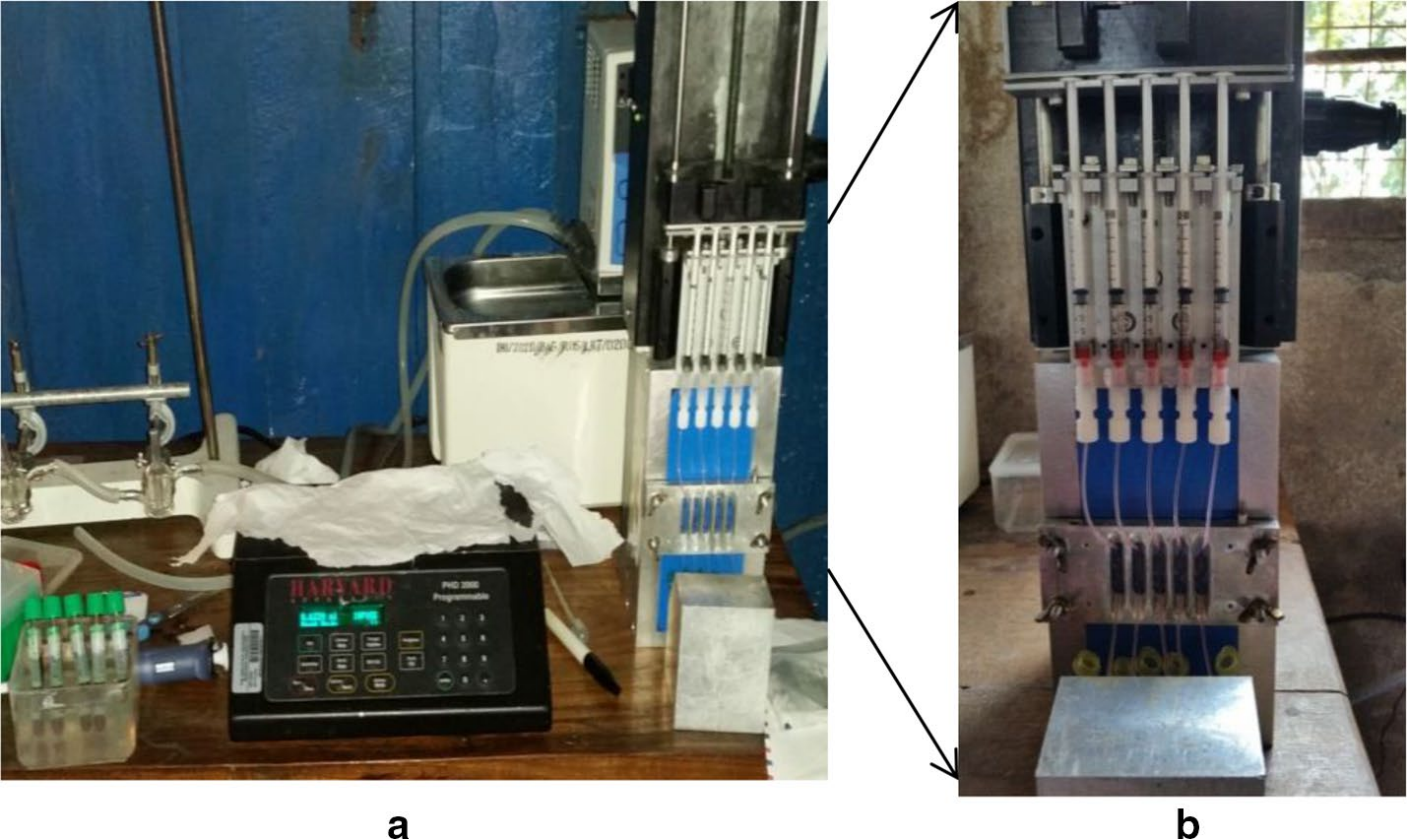
MDM apparatus used in the field study. **a** Note five-channel sample flow arrangement operated by a syringe pump and a programmable controller. The flow channel assembly is pressed against the permanent magnet (*painted blue*) using four wing nuts. **b** Five blood samples visible inside 1 mL syringes prior to being pumped back into to the collection tubes (*bottom*) through the flow channel pressed against the magnet. The weight of the system is estimated at less than 3 kg

The MDM assay was performed as previously described by [6] with modifications. The principle of MDM operation is shown in Fig. 1. Prior to its being shipped to Tanzania from the Cleveland Clinic, Lerner Research Institute, USA, it was tested and validated with the laboratory *P. falciparum* strains, 3D7 and NF54 at Case Western Reserve University. In the course of those preliminary studies a protocol was developed suitable for the planned field operation, and was tested for the intra-erythrocytic parasite enrichment by the MDM assay. In brief, the MDM apparatus as described in [6] was modified by equipping it with a programmable syringe pump (PHD 2000 Programmable, Harvard Apparatus Inc.) for automatic sample aspiration and infusion (back to the sample container), resulting in a 1.5× increase on average of the time of blood sample exposure to the magnetic field compared to single pass operation, and thus increasing the likelihood for the magnetic cell capture. The device was designed to process five different samples in a parallel fashion (Fig. 2), at approximately 10^8^ cell/mL number concentration, volume 0.5 mL each, with the flow rates set at 0.026 mL/min for aspiration and 0.013 mL/min for infusion, resulting in 42 min of total processing time of five different samples from five different individuals. Importantly, MDM concentrates infected RBCs on one area on the slide to increase sensitivity and decrease the time to read slides compared to LM. The MDM apparatus was a laboratory prototype but its principle of operation (magnetic separation in microfluidics channels) makes it amenable to miniaturization for field operation.

Briefly, blood samples were diluted using PBS (pH = 7.2) in a ratio of 1:10 before pumping 500 µL of each test sample through the flow channels. Infected RBCs were collected and concentrated on a 0.13 mm-thick Mylar sheet (also known as polyester slide) by the magnet to ensure maximum late parasitic stage enrichment from five samples obtained from the five different flow channels. The blood samples for the MDM analysis were prepared and processed freshly in the field. Cells captured on the Mylar slide were fixed by 100 % methanol for 30 s and stained with 10 % Giemsa for 30 min. Once the slides dried, they were observed and analyzed under a light microscope at 100× magnification.

### Data analysis

Data were entered and processed in Microsoft Access (Microsoft Corporation, Microsoft Way- Redmond, Washington) and analysed in STATA 11 (StataCorp, College Station, Texas). Data were entered in 2 × 2 tables and analysed for sensitivity (the probability that the assay will be positive when the infection is present) and specificity (the probability that the assay will be negative when the infection is absent) using the following formulae:

1 $${\text{Sensitivity}} = \frac{TP}{TP + FN} \times 100 \, \%$$

2 $${\text{Specificity}} = \frac{TN}{TN + FP} \times 100 \, \%$$

where *TN* represents true negative, *TP* true positive, *FN* false negative and *FP* false positive. The agreement between LM, RDT, and MDM were determined using STATA 11. The data sets were compared pair-wise by selecting one of the methods as a reference standard for TN and TP determinations (LM v. MDM, and MDM v. RDT).

## Results

### In vitro results

**Fig. 3:**
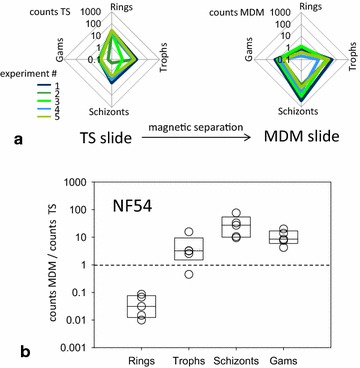
MDM counts increase sensitivity to late stage parasite forms and gametocytes (NF54 strain). **a** Radar log-plot of Thin Smear (TS) and MDM mean counts from five independent experiments showing increase in the late stage and gametocyte content on the MDM slides relative to TS slides. **b** Fold enrichment of infected RBCs (iRBCs) counts on the MDM slides relative to TS slides showing individual mean counts (*circles*) and quartile boxes (unity line indicates no-enrichment limit). The increase in fold enrichment with the parasite stage is consistent with the increase in intraerythrocytic haemozoin concentration [7]

**Fig. 4:**
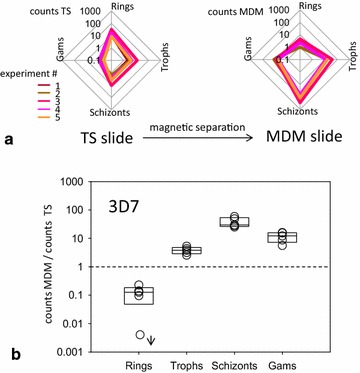
As in Fig. 3, MDM counts increase sensitivity to late stage parasite forms and gametocytes for 3D7 strain. Note: the same trend as in previous Figure. The *arrow* indicates null counts on MDM slide for that particular experiment

The slides were counted against 2000 total RBCs under the microscope. Late stage parasites, trophozoites, schizonts, and gametocytes were enriched by MDM compared to thin smear counts, schizonts being the most observed developmental stage of the parasite on MDM slides compared to thin smear (TS) slides (Figs. 3, 4). The average corresponding parasitaemia density on the MDM slide and thin smear was 164.6 parasites/µL and 26.5 parasites/µL, respectively. The gametocytes enrichment by MDM for NF54 and 3D7 *P. falciparum* strains compared to TS slides was at approximately 10-fold.

### Field results

Three methods, LM, MDM and RDT were compared to detect *P. falciparum* infections in 303 school children. Amongst the *P. falciparum* positive individuals as detected by LM, RDT and MDM, 88.2, 93 and 89.8 %, respectively, had body temperature below 37.5 °C. However, body temperature had no statistical significant influence on the proportion of *P. falciparum* infections among the asymptomatic individuals.

The results are summarized in Tables 1, 2. Table 1 shows prevalence of *P. falciparum* for all stages by all three tests (LM, RDT and MDM) and gametocytes prevalence (available from LM and MDM only). The prevalence of *P. falciparum* infections by MDM, RDT and LM were 19.5 % (95 % CI 15.2–24.4), 17.2 % (95 % CI 13.1–21.9) and 7.6 % (95 % CI 4.9–11.2), respectively. The difference between MDM and LM in detecting *P. falciparum* infections was highly significant (p < 0.01), while there was no significant difference when MDM was compared with RDT (p = 0.3748). The MDM results were further analyzed by sensitivity and specificity (Eqs. 1, 2) against the method that showed the highest prevalence of infection. The results are shown in Table 1. Thus the LM sensitivity was relatively low 36.5 % (95 % CI 32.2–60.5) while that of RDT was better than that of LM, 62.5 % (95 % CI 49.5–71.8) compared to MDM. Interestingly, the MDM specificities with respect to LM and RDT to all forms of *P. falciparum* were high, 87.5 % (95 % CI 71.2–89.6 %) and 89.0 % (95 % CI 82.9–91.4), respectively.

**Table 1:** Prevalence of *Plasmodium falciparum* all stages as determined by three diagnostic tests (LM, RDT and MDM) and gametocytes prevalence determined by LM and MDM only, in both cases n = 303

	LM (%)	95 % CI	RDT (%)	95 % CI	MDM (%)	95 % CI
All stages	23 (7.6)	4.9–11.2	52 (17.2)	13.1–21.9	59 (19.5)	15.2–24.4
Gametocytes	4 (1.3)	0.4–3.3	–	–	12 (4)	2.1–6.8

**Table 2:** Comparison of sensitivity and specificity for the parasite detection between MDM, LM (part A1) and RDT (part A2) and comparison of sensitivity and specificity of gametocytes detection between MDM and LM (part B)

A1	MDM				
LM	Positive	Negative	Total		(%)
Positive	19	4	23	Sensitivity	36.5
Negative	40	240	280	Specificity	87.5
Total	59	244	303		

A2	MDM				
RDT	Positive	Negative	Total		(%)
Positive	28	24	52	Sensitivity	62.5
Negative	31	220	251	Specificity	89.0
Total	59	244	303		

B	MDM (gametocyte)				
LM	Positive	Negative	Total		(%)
Positive	1	3	4	Sensitivity	26.7
Negative	11	288	299	Specificity	96.5
Total	12	291	303		

The prevalence of *P. falciparum* gametocytes was 1.3 % (4/303 95 % CI 0.4–3.3) by LM but was considerably higher and equal to 4 % (12/303 95 % CI 2.1–6.8) by the MDM (Table 1). The difference between the two methods in detection of gametocytes was statistically significant (p = 0.008). The sensitivity of the MDM to gametocyte detection was low 26.7 %, (95 % CI 0.6–80.5) but its specificity was high, 96.5 % (95 % CI 93.7–99.1) indicating that both tests do not differ significantly in specificity of gametocyte detection (Table 2B).

## Discussion

This study demonstrates that MDM could successfully detect sub-microscopic parasitaemia from asymptomatic school children. Overall, MDM showed an improved detection rate for the gametocytes by threefold compared to LM, important in areas where malaria is declining and gametocyte density has become a variable of interest [22]. Sub-Saharan Africa has experienced a marked reduction of *P. falciparum* infection rates and parasitaemia levels, a situation that poses a serious challenge to the conventional malaria diagnostic tools, such as LM, to clearly estimate malaria burden [28]. The MDM device from this study improved detection of parasitaemia and gametocytaemia compared to LM. This is not surprising as the MDM device used in this study was a modified version of the previous one used in PNG [8, 9], by using two-directional flow across the magnet for increased capture of iRBCs and it offers a convenient way on set up and operation for a day-to-day use.

The increased rate of gametocyte detection by MDM compared to LM is related to two factors: depletion of uninfected RBCs from the slide by the fluid flow and concentration of the infected RBCs (iRBCs) by the magnetic field in a small area of the slide covered by only a few microscope fields of view. In comparison, the LM preparation is smeared over a substantial part of the microscope slide surface and the iRBCs are diluted by the uninfected cells in the microscope field of view. This MDM deposition pattern can assist microscopists in locating iRBCs on the slide, hence contribute to more rapid evaluation of blood slides and reduction of slide reading time. Interestingly, the gametocyte enrichment rate (relative to LM) in vitro, 10× was higher than that determined for the asymptomatic children (3×). The responsible mechanism could be higher viscosity of the diluted blood from human subjects compared to the viscosity of the iRBCs culture media (not measured) increasing the loss of iRBCs from MDM slides. As MDM provides mature gametocyte counts in a given blood volume (50 µL), this enumeration strategy may help in studying the relationship between gametocyte density and mosquito infectivity. A number of field studies suggested that microscopic and sub-microscopic gametocytes from humans are infectious to mosquitoes [16, 29, 30]. Additionally, because MDM captures intact iRBCs on the microscope slide, it provides a convenient platform for cytochemical differentiation of species, species identification and stage-specific morphologic features analysis of the parasite.

This study involving asymptomatic school children from a low malaria transmission setting confirmed preliminary results from in vitro work showing that late stage parasites, microscopic and sub-microscopic gametocytes were susceptible to MDM capture. The results show that MDM increased detection of *P. falciparum* infection by 2.5-fold (19.5 % vs 7.6 %) and was more specific (87.5 %) over LM and RDT (89.0 %), although its sensitivity was almost similar to RDT. Comparable findings were reported in other studies [6, 8, 9, 31] although blood samples used in this study were from asymptomatic school children who are potentially reservoirs of gametocytes something which differs from other studies. Similarly, MDM detected three times more gametocytes compared to LM what indicate that *P. falciparum* gametocytes were usually underestimated by the current diagnostic tool, LM. Malaria infection underestimation by current diagnostic tools was also observed by the studies conducted in southeast Tanzania [32]. Moreover, several other studies emphasized on the importance of detecting sub-microscopic gametocytes that occur below the detection limit as they perpetuate transmission when ingested by a female mosquito [17, 30, 33].

A significant challenge on management of malaria disease is an easy way of obtaining a reliable, simple and sensitive diagnosis. In many endemic areas LM remains the most available method [34] while in Tanzania, LM and RDT are the widely used as standards for malaria parasite confirmation and detection methods respectively. However, RDT has been used as a quick diagnostic test although its detection capacity is limited to ≥200 parasites per microlitres [10] and it is incapable of distinguishing sexual and asexual forms of the parasites. The familiarity with the LM use in the field, the higher sensitivity of MDM to gametocytaemia detection than that of LM (although operating on the same principle of microscopic examination) together with its ability to distinguish between sexual and asexual forms makes it an interesting potential addition tool in detecting sub-microscopic parasitaemia and accurately estimating the malaria burden in low malaria transmission settings. Moreover, the estimated equipment cost could be reduced to that of a commercial, high gradient magnetic separation (HGMS) column system in multiple column configuration but with significant savings on disposables as the material cost of MDM slides is cheaper compared to multiple US dollars for one HGMS column [9].

The MDM instrument used in this study was a laboratory prototype and, therefore, lacking the user-friendly features of operation. The study served an important purpose of identifying those features that would require simplification before the MDM device could be adopted for wider use in field studies. The other important consideration is that it relies on the presence of haemozoin to magnetically concentrate iRBCs on the slide leaving out uninfected RBCs, therefore absence of uninfected RBCs on the slide, increases detection of iRBCs.

The current instrument requires AC power for the syringe pump operation, conveniently supplied by DC-AC inverter or 12 V automobile battery. These could be miniaturized by taking advantage of increasing availability of small pumps for point of care laboratory medicine, including USB powered pumps (powered by laptop computer batteries) and solar panels or completely eliminated by redesigning the flow path for gravity-fed operation. The combination of magnetic separation and microfluidics of the MDM prototype coupled with its ability to distinguish low-level parasite stages, particularly gametocytes, makes it an ideal candidate for miniaturization to further reduce size, weight and minimize or eliminate power consumption. This improvement could make the instrument more user-friendly, cost-effective and hence attractive as a tool for malaria transmission in the laboratory and in the field.

## Conclusions

MDM enhanced detection of sub-microscopic *P. falciparum* infections and estimation of gametocyte density compared to LM and the results were comparable to those determined by RDT. Gametocyte enrichment by MDM was threefold higher than LM, offering improvements in results turn-around time and detection of sub-microscopic gametocytes, a feature which cannot be accorded by RDTs. Rather than depending on blood smears alone, the reliable, simple and field-adaptable MDM system could be useful to investigators and clinical researchers working on transmission studies as well as routine diagnosis after modification and miniaturization. On the other hand, these findings could further provide information on gametocytes density levels from asymptomatic individuals that become infectious to mosquitoes in settings where malaria transmission is substantially decreasing.
